# Construction Notes

**Published:** 1892-11-26

**Authors:** 


### CONSTRUCTION NOTES.

On October 19th Professor Alexander Ogstan opened the new hospital at old Machar Poorhouses, Aberdeen. It is to be used exclusively for the treatment of pauper patients. The building, which cost about £2,000, has a frontage of 160 feet.

A new disinfector-house and laundry have just been added to the Falkirk Fever Hospital. The new buildings were opened on October 13th, by Mr. Learmouth, convener of the Hospital Committee.

The new central offices of the Belfast Dispensary are situated in Glengall Street, and were opened on November 8th, 1892. On the ground floor are to be found the usual offices assigned to the daily work of the dispensary. The board room is upon the first floor of the building, and adjoining it are several consulting rooms, and other apartments for the use of the medical staff. The architects were Messrs. Young and Mackenzie, the builder Mr. William Kerr, and the furniture was supplied by Messrs. J. C. Mayrs.

